# A Qualitatively-Informed Approach to Examining the Construct Validity of Alcohol-Involved Sexual Violence

**DOI:** 10.1007/s12119-025-10324-5

**Published:** 2025-01-29

**Authors:** Tiffany Chiu, Nili Gesser, Benjamin W. Katz, Ellei Burmeister, RaeAnn E. Anderson

**Affiliations:** 1 Department of Education, Health, and Behavior Studies, University of North Dakota, Education Building Room 200, 231 Centennial Drive Stop 7189, Grand Forks, ND 58202-7189 USA; 2 Department of Psychology, College of Arts and Science, University of North Dakota, Grand Forks, ND USA; 3 Department of Psychology, College of Arts and Science, University of Wisconsin-Milwaukee, Milwaukee, WI USA; 4 Alluma, Crookston, MN USA

**Keywords:** Sexual violence, Cognitive interview, Qualitative, Sexual assault, Alcohol-involved rape, Consent

## Abstract

One in five women experience sexual victimization in her lifetime, making sexual victimization a significant public health crisis. This public health crisis also has detrimental health consequences. Understanding the scope of sexual violence and the effects of prevention programming requires accuracy in measuring sexual violence. Yet, a wealth of research suggests lack of precision in sexual violence measurement and potential confusion among respondents in how to interpret items on sexual victimization and perpetration questionnaires. Specifically, alcohol-involved sexual violence questions are among the hardest to operationalize in such questionnaires, due, in part to cultural ideas about alcohol and idiosyncratic effects of alcohol on behavior. The current study addressed this gap by assessing the construct validity of alcohol-involved sexual violence questionnaire items. We used a cognitive-interviewing qualitative approach to analyze interviews with forty participants to better understand the cognitive and interpretive language nuances between “took advantage of while drunk” and being “too drunk to consent.” Qualitative thematic analysis was conducted to identify and summarize differences in how stimuli were perceived and interpreted. Our thematic analysis identified three themes: (a) respondents used behavioral indicators as an indicator of impaired decision-making, (b) respondents perceived and expressed complexity in understanding alcohol-related consent, and (c) some respondents attributed the differences between the phrases to reflect experiences of either perpetration and victimization. The results underscore the importance of refining questionnaires using behavioral indicators of alcohol intoxication to differentiate between incapacitated and impaired alcohol-involved incidents, clarifying non-consent language, and being purposeful in describing a state or an action related to the consumption of alcohol, to better measure alcohol-involved sexual violence.

## Introduction

Sexual violence, defined as a nonconsensual sexual experience, affects at least one in five American women and nearly as many as men (Black et al., 2011; Stemple & Meyer, 2014). Currently, the Center for Diseases Control and Prevention defines rape as, “a type of sexual violence that involves unwanted penetration of a victim through physical force or when the victim is unable to consent due to drugs or alcohol”. Sexual violence can impart a range of long-lasting and highly variable negative health consequences including depression, substance use, posttraumatic stress disorder as well as relationship impairment and lower occupational attainment (Byrne et al., 1999; Dworkin et al., 2017; Koss, 1993).

Alcohol is involved in 75% of rapes (Koss et al., 2022). Alcohol use and the social norms, expectations, and consequences of drinking are gendered in Western societies, including in the context of sexual violence (Frank et al., 2020). For example, victims of alcohol-involved sexual victimization are more likely to be blamed by others and blame themselves for sexual victimization in comparison to victims of sexual victimization where alcohol was not involved (Littleton et al., 2009; Sims et al., 2007). Indeed, Koss et al., (2022) speculate that the increase in prevalence of rape in their analysis of large scale data collected in 2015 compared to 1985 is the result of gendered response patterns and better operationalization of alcohol-incapacitation tactics.

Despite the alarming rates of alcohol-involved sexual victimization and associated consequences, alcohol-involved victimization may be one of the most difficult types of rape to operationalize, as alcohol-related effects on consent exist on a continuum, partially due to the idiosyncratic pharmacological, psychological, and behavioral effects of alcohol (Ward et al., 2012). For example, body weight and composition can affect the intensity and duration of alcohol's effects, while metabolism and enzyme activity determine the rate of alcohol breakdown; these physiological processes operate differently in biologically male vs. female bodies. Psychological and behavioral factors, such as mood and expectations, further influence the idiosyncratic effects of alcohol intoxication and can do so in gendered ways (Frank et al., 2020; Wolkowicz et al., 2019). Thus, the goal of this study was to qualitatively examine how young adults of any gender interpret different common survey phrases related to alcohol-involved sexual violence, in order to improve the measurement of alcohol-involved sexual violence.

### The Variability of Alcohol-Related Sexual Violence and Measurement Challenges

Prior research has utilized diverse approaches to operationalize alcohol-involved sexual violence, reflecting significant variability (Anderson et al., 2021a, b; Koss et al., 2022). This variation is fitting, as it allows for capturing the numerous ways alcohol may play a role in sexual violence. Theory suggests that measures of sexual violence should encompass three key elements: consent, tactics, and sexual behaviors (Cook et al., 2011). Within this framework, alcohol can serve as a deliberate tactic of perpetration, such as encouraging someone to drink, secretly providing stronger-than-expected drinks, or spiking drinks with drugs. Alternatively, it can function as an opportunistic tactic, such as targeting someone who is already intoxicated through verbal pressure, or taking advantage of someone who is incapacitated. Operationalizing just these five examples would result in five distinct items, even when considering alcohol solely as a tactic. Factoring in how alcohol impacts consent adds further complexity, as consent is itself a nuanced and dynamic process (Muehlenhard et al., 2016).

In addition to differences in alcohol metabolism by biological sex (Thomasson et al., 1995), alcohol use expectancies and context of use expectancies vary by gender and other factors such as sexuality, race, and income (Nagata et al., 2023; Kuntsche et al., 2006; Wolkowicz et al., 2019). These differences can create difficulty in measurement in that measures may need to overcome beliefs and stereotypes about alcohol that respondents hold. For instance, women who consumed alcohol consensually may be less likely to endorse an item such as “taken advantage of while drunk” due to internalized rape myths—false beliefs about who is raped and under what circumstances—that influence how they interpret their experiences (that is, they do not see themselves as being “taken advantage of” if they drank voluntarily).

Another challenge to properly measuring alcohol-involved sexual violence relates to the difference between incapacitation and impairment due to alcohol consumption. This distinction is critical for measurement, as these states have different implications for a person's ability to provide consent and fall into distinct legal categories. Impairment refers to a reduction in functional ability, such as difficulty walking, whereas incapacitation indicates a complete inability to perform a task, such as being unable to walk at all (McConnell et al., 2017). Incapacitation encompasses a range of states that include but are not limited to unconsciousness (National Cancer Institute, 2025).

Variation in and difficulty with measuring alcohol-involved incidents may contribute to imprecise or incorrect conclusions about the nature of alcohol-involved sexual violence if impairment and incapacitation are conflated. McConnell et al. (2017) demonstrated how using a dichotomous alcohol variable (i.e., yes/no) to capture victimization could obscure differences between assaults in which the victim was unconscious because of alcohol, and assaults in which the victim was only impaired. This study found that patterns of PTSD symptoms varied across the groups. Specifically, individuals who experienced incapacitated assaults exhibited greater rape acknowledgment— defined as the decision to endorse and label their experience as rape (Anderson et al., 2021a, b)—compared to those who were impaired, who reported lower acknowledgment and higher levels of peritraumatic fear. Littleton et al. (2009) similarly found differences between those who were impaired vs. those who were incapacitated by alcohol such that those who were incapacitated reported greater feelings of stigma related to the assault. Thus, not only are these nuances important in reflecting the reality of alcohol, but they also have clinically meaningful differences for post-assault intervention.

### Measurement Items in Sexual Violence Surveys

The current study focused on instances, asked about in questionnaires, where alcohol contributes to the vulnerability of the victim. Such items address, inter alia, instances where people who perpetrate appear to intentionally target women with sex-related alcohol expectancies, exploiting this as a potential vulnerability (Benson et al., 2007). Our study did not relate to instances where individuals may intentionally use alcohol to enhance consensual sexual experiences and should have the freedom to do so safely (Celio et al., 2016; Benson et al., 2007). College students may recognize their right to use alcohol in this context and understand that their ability to consent can vary with consumption levels; however, perceptions and norms about how much alcohol impairs consent differ widely (Jozkowski et al., 2023).

The measurement of alcohol-involved incidents and incapacitation varies widely across sexual violence surveys (see Table 1). For instance, different versions of the Sexual Experiences Survey (SES) demonstrate varying emphases on incapacitation. The 2007 SES (Koss et al., 2007) uses the phrasing, “Taking advantage of me when I was too drunk or out of it to stop what was happening,” whereas the 1985 version included, “made a woman have sexual intercourse when she didn’t want to because you gave her alcohol or drugs.” Koss et al. (2022) suggest that the latter phrasing is a less effective measure. Other tools, such as the Sexual Aggression Victimization and Perpetration Scale (SAV-S), center on alcohol’s impact on resistance, using language like, “by exploiting the fact that you were unable to resist (e.g., after you had had too much alcohol or drugs)” (Krahé et al., 2015).

**Table 1:** Examples of alcohol-involved items on sexual violence questionnaires

Questionnaire	Alcohol-involved operationalization	Reflects a continuum?	Operationalizes incapacitation?
The sexual experiences survey 2007	Taking advantage of me when I was too drunk or out of it to stop what was happening	No	Yes
Sexual aggression victimization and perpetration scale	By exploiting the fact that you were unable to resist* (e.g., after you had had too much alcohol or drugs)	No	Yes
Post-refusal sexual persistence scale	They took advantage of the fact that you were drunk or high	Yes	No
National intimate partner and sexual violence survey	When you were drunk, high, drugged, or passed out and unable to consent….had vaginal sex with you?	Yes	No
Sexual experiences survey 1987	Had a man have intercourse with you by giving you alcohol or drugs?	Yes	No

In this case, incapacitation is defined as being unconscious

^*^Original bolding

In contrast, other approaches assess alcohol involvement separately, first identifying sexual violence incidents and then asking participants whether and how much they had been drinking during the event. This variability in measurement highlights the complexity of operationalizing alcohol-involved sexual violence, particularly in distinguishing between tactics and consent.

### Impacts of Measurement Challenges

These aforementioned measurement challenges are reflected in reliability estimates. In examining test–retest reliability, Anderson et al., (2021a, b) found poorer reliability for alcohol-involved incidents compared to physical force incidents. Gender differences further complicate the issue, with reliability estimates being stronger for men than for women, underscoring the unique complexities of alcohol-involved vulnerabilities. Similarly, Anderson and Delahanty (2019) also observed gender differences in alcohol-involved victimization incidents, noting that the agreement between two different questionnaires in identifying cases was stronger for men than for women. The authors hypothesized that this might be due to gender differences in alcohol metabolism, which could make it easier for men to recognize and report self-impairment, whereas women may find it more challenging to identify an item that accurately captures their experience.

Adding to these challenges, a systematic review of perpetration measures and their use highlighted the frequent addition of alcohol- and substance-use-related items as one of the most common modifications, reflecting the lack of comprehensive, standardized measures for alcohol-related sexual violence (Anderson et al., 2021a, b). Despite these challenges and their impact on measurement results, prior research has primarily focused on quantitative evaluations of these measures (e.g., Anderson et al., 2017; Struckman-Johnson et al., 2019), often overlooking alcohol-specific violence and the nuances of how respondents differentiate between alcohol-related survey items.

### Need for Qualitative Research

Quantitative studies focused on examining the validity and reliability of the SES and related measures (Anderson et al., 2018; Johnson et al., 2017) have highlighted the need for qualitative methodologies to better clarify nuances, particularly in how respondents interpret researcher-designed phrases (Anderson et al., 2021a, 2021b). For example, Strang et al.’s (2013) study found that men's interpretations of non-consent language is highly variant, and the number of acts of aggression reported depended on the specific consent language used in the survey: "without her consent" versus "after she initially said 'no'." The researchers suggested that further research is required to explore the impact of language specificity across various acts.

Gendered differences in interpretations of identical survey content were also documented by Strang et al., (2013), and Jeffrey and Senn (2024), who found that womens’ descriptions of verbal coercion experiences were often harsher and more indicative of severe intimate partner violence in comparison to mens’. A recent thematic qualitative analysis examined the content validity of verbal coercion and whether respondents distinguish coercive insistence from consensual compliance (Welk et al., in press)—one of the most context-dependent and heterogeneous forms of violence.

Cognitive interviewing is a particularly helpful qualitative technique for developing survey and questionnaire items. The goal of cognitive interviewing is to identify potential problems with survey questions, by understanding how participants interpret specific instructions, wording, and phrases (Beatty & Willis, 2007). Participants are asked not only to respond to the stimuli, but also how they arrived at their responses. One form is the classic Think-Aloud paradigm wherein participants are asked to verbalize their thoughts (e.g., cognitive processes) as they are presented with item stimuli (Davison et al., 1997). Applications of this classic technique from the survey literature to violence research has been fruitful but under-utilized.

One of the few sexual violence questionnaires to have demonstrated cross-country validity is the "Sexual Aggression and Victimization Scale, (SAV-S), a psychological tool used to assess the prevalence of sexual aggression victimization and perpetration. Using a cognitive interviewing paradigm, participants of multiple genders were presented with items and asked to “imagine/think about the kind of scenario they would have in mind in order to make a decision on whether to respond “yes” or “no” (Krahé et al., 2015). Responses were deductively coded for functional equivalence to assess validity across the ten participant countries and cultures. Another example of cognitive interviewing is a pair of mixed methods studies, which found that a partner violence questionnaire instruction set that included a qualifier phrase (e.g., "do not include joking around in your response") designed to decrease spurious reports of physical violence did not significantly affect the reporting rates of victimization (Sargent et al., 2020; Yule & Grych, 2022). The qualitative findings of that study revealed the mechanism for this quantitative finding: Respondents were more influenced by the way behaviors were described in the item than by the instructions provided, and so they were unable to disregard certain behaviors as the instructional qualifier intended. The findings from these two studies suggest that the development of violence surveys should prioritize clear and precise descriptions of behaviors to improve the consistency and reliability of sexual violence measurements. This is particularly important when aiming to accurately and comprehensively identify alcohol-related violence.

### The Current Study

The goal of the current study was to examine how young adults of all genders interpret different operationalizations of alcohol-involved sexual violence to improve measurement of this common form of sexual violence. After reviewing the literature, we focused on two common operationalizations of consent in alcohol-related sexual violence. The first operationalization, “took advantage of while drunk,” reflected a possible continuum of alcohol impairment, and was taken from the SES and Post-Refusal Sexual Persistence Scale (PRSPS).We also tested a more precise version, “too drunk to consent,” which was designed to more clearly indicate incapacitation, in line with the CDC’s survey operationalization (see Table 1). Notably, “took advantage” language is used in both the victimization and perpetration forms of the SES and PRSPS, which are some of the most commonly used measures (Anderson et al., 2021a, b).

## Methods

### Participants and Recruitment

Forty young adults were recruited to participate in our semi-structured cognitive interviews between August to December, 2020. Recruiting young adults across genders and without respective to their own victimization/perpetration history is consistent with guidelines for cognitive interviewing protocols (Groves et al., 2011), the use of the study stimuli in prior research, and prior violence measurement research using this and similar methods (Krahe et al., 2015; Jeffrey & Senn, 2024; Sargent et al., 2020). The study was described as “Help scientists out: Does this make sense? How should we ask about sexual assault?” on social media flyers (e.g., Facebook, Instagram, and Twitter) and in the Psychology Department subject pool (e.g., SONA). Researchers also actively recruited community participants and subpopulations (e.g., LGBTQ people) via relevant listservs. Participants were eligible if they were 18 or above. In the absence of established guidance on an appropriate sample size for cognitive interviews, we aimed for and achieved recruitment of 40 participants as an estimate from other previous studies (Luckett et al., 2022).

The average age of the participants in the study was 22, with a range of 18–34 years old. Participants identified primarily as female (60%) and White (87.50%), Native Americans (7.50%), and Asian/Pacific Islander (5.00%). The sample was majority non-Hispanic (92.50%). Regarding sexual orientation, the largest group (77.50%) identified as heterosexual, while 12.5% identified as bisexual, 2.5% as gay or lesbian, 5.00% as queer, and 2.50% provided no response. When asked about their family income, 45.0% of the respondents reported a family income of $100,000 and above, with 17.5% falling in the range of $40,000 to $59,999, and 12.0% were unsure of their family's income. In terms of relationship status, 30.77% of the respondents were single, 48.72% were in a relationship with one partner, 7.69% were married to one partner, and 2.56% were in a polyamorous relationship without being married. The majority of participants (81.9%) had higher education (some college or above), while 18.0% did not (high school or technical college).

The interviewers were students under the supervision of the Principal Investigator. The team included nine graduate and undergraduate women who varied in racial identity (i.e., White and Indigenous), socioeconomic status, sexual identity (i.e., bisexual and heterosexual), and region they were raised in (i.e., Mountain, West, and Midwest locations).

### Procedure

The study was approved by the Principal Investigator’s Institutional Review Board [masked for review]. Each cognitive interview was recorded on Zoom and lasted on average 70 min. After completing the study, participants were compensated with either course credit or a $20 gift card. Prior to administering the cognitive interview on zoom, researchers emailed participants a Qualtrics survey link which contained a consent script, and participants affirmed their consent to the interview by both clicking a button and verbally conveying their decision to the interviewer before the interview began online. Participants are initially asked to complete the survey questions independently in Qualtrics on their own device before the study session. However, if a participant does not have enough time to respond in Qualtrics prior to the cognitive interview session, the interviewer will share their screen via Zoom to display the survey questions and guide the participant through the responses. The interviewer will then record or transcribe the participant’s answers as needed. A semi-structured interview protocol was employed to encourage participants to compare and contrast items, while allowing interviewers to ask clarifying follow-up questions. The items in this qualitative interview protocol, which were derived from the SES and PRSPS, were part of a larger cognitive interview. The interview prompts of focus in this study were:

- What is the difference between “took advantage of while drunk” and “too drunk to consent”?
- How can you tell if someone is too drunk to consent?
- How did you come up with your answer?

### Data Analysis

Four researchers, led by the second author, analyzed the interview transcripts thematically. Interview transcripts were de-identified prior to coding and coders did not have access to respondents’ demographics (e.g., gender, race, etc.) in order to minimize bias and protect respondents confidentiality during coding. We first conducted a preliminary analysis of the transcripts by reading through the completed transcripts and coding them line-by-line to reveal important themes. We then met to discuss the coding and developed an agreed-upon codebook. We used an inductive approach to coding, utilizing the data to interpret our results (Thomas, 2006). We used Excel to organize and code the same segments in the data and then reviewed our coding to improve inter-rater reliability until 80% agreement among coders was reached (Kurasaki, 2000). Following the initial coding process, our team engaged in thorough discussions to reconcile any differences that emerged. Through this collaborative process, we achieved a shared understanding of the data and a framework of higher-level themes, detailed below, that arose from the coded data.

## Results

Overall, we found that participants reported a range of personal perspectives and experiences regarding the different operationalizations of alcohol-involved sexual violence items (e.g., What is the difference between “took advantage while drunk” and “too drunk to consent?”). Our analysis identified three main themes: (a) behavioral markers were used by respondents as indicators of impaired decision-making, particularly in distinguishing between individuals who are incapacitated and unable to provide consent versus those who may still retain some decision-making ability, (b) some respondents used ambiguous terms to talk about consent in the context of the two phrases, potentially reflecting the complexity in identifying consent in the different phrases, and (c) participants distinguished between the two phrases as indicating either a state or an action, and specifically a state of victimization versus the action of a perpetrator. We elaborate on these three themes below.

### Behavioral Indicators of Lack of Ability to Consent

Respondents in the study noted two forms of markers for impaired decision-making in the context of consent: behavioral and emotional, with the majority (73%) embracing behavioral markers, and in particular, slurred speech. For example, a participant noted that the language of these behavioral signs includes the following:

“[Individuals are] acting erratic and don’t speak clearly. They’re not initiating it [sexual contact]… they don’t want it. Usually when it’s consensual it's something on both parties that they engage together, usually if one is too incoherent to give consent it’s just a one-party deal.” (Participant 209)

The participant's observation draws attention to the fact that when discussing behavioral (e.g., physical) indicators related to consent, the language commonly used may reflect the understanding that people displaying these behaviors are too impaired to engage in consensual sexual behaviors.

Similarly, Participant 235 emphasized behavioral markers that indicate when someone is too drunk to consent including, “not walking straight, [not] thinking straight, [not having] common sense, talking,…and driving…. I remember how I was acting when I was drunk and couldn’t think straight. You shouldn’t be anywhere alone or drive because you can’t take care of yourself.” These indicators illustrate various levels of impairment that can affect decision-making abilities, highlighting the importance of distinguishing when impairment transitions into incapacitation. Likewise, another participant said that people are too drunk to consent when they are “unable to form a sentence, slurring words, trouble walking, and bad motor control” (Participant 220). This participant noted that behavioral functioning can become impaired, including their verbal communication skills. As a result, the ability to express themselves clearly and decode verbal and non-verbal cues from others may be compromised due to alcohol.

Along the same lines, Participant 208 gave a detailed list of physical markers that indicate when someone is too drunk to consent:

If they can’t verbally consent, that’s usually a starting point. If their judgment is impaired enough that they would do other things or say other things that would make you question their judgement, they’re probably not able to consent. If they are exhibiting physical signs of being drunk like swerving around or slurring their words, probably a sign that they’re too drunk to have informed consent unless you had some sort of prior understanding that this person had those types of actions while maintaining good judgement.

Although this participant mentioned impaired judgement in the context of alcohol consumption, they relied on physical behaviors of the impacted person to judge whether they were too drunk to consent.

While most participants emphasized the behavioral influence of alcohol, one participant also related to the emotional impact of alcohol on behavior alongside physical markers: “… They are acting differently emotionally than they typically would be, like really sad or really angry. We’re more prone to express emotion when we’re intoxicated” (Participant237). This participant acknowledged the specific role of negative emotions exacerbated by alcohol and how this may interfere with the ability to consent. As shared by this participant, changes in emotional distress, including agitation and aggression, may interfere with one’s ability to consent. Whether they emphasized emotional or behavioral markers, nearly half of the respondents reported that they drew on their own experience when intoxicated to claim that alcohol can affect language used, everyday motor skills, behavioral abilities and emotions when consent is given in the context of alcohol consumption.

### Complexity in Manifesting Consent

While most participants (85%) acknowledged that “took advantage of while drunk” was different from “too drunk to consent”, some participants expressed challenges in articulating the distinctions between the two phrases, potentially reflecting the blurry line that exists between being only impaired to being fully incapacitated by alcohol when giving consent. For example, one participant noted:

I think the difference is **a very rough line** to see especially if they are drunk. When they are too drunk to consent, they lost a heavy percentage of coherency, personality, no memory of recent event, or inhibitions, you are **probably** past that point to give full consent (Participant 222, emphasis added).

This participant used nuanced terms such as “rough line” and “probably” to explain the moderating influence of alcohol on consent and indicated the second scenario (“too drunk to consent”) as more indicative of lack of consent.

Another participant also used ambiguous terms while noting that alcohol incapacitation is a barrier to verbally communicating about sexual consent:

Took advantage of **probably** when they were conscious and were able to verbally say things. Too drunk to consent means they **could be conscious or unconscious** and could not consent because they were incoherent **maybe** (Participant 211) (emphasis added).

In other words, for this participant too, “too drunk to consent” is more indicative of incapacitation than “took advantage of.”

Participant 229 was more explicit in aligning incapacitation with being too drunk to consent, saying:

Took advantage of could mean that someone gave consent and then was pushed past that consent and too drunk is a violation of whatever sex act was performed and represents an incapacitation and whatever it means to consent to that.

These two participants claim that the phrase “took advantage of while drunk” is less indicative of non-consent than the phrase “too drunk to consent,” which is more unequivocal.

Participant 201 also provided a distinction between the phrases "took advantage of while drunk" and "too drunk to consent" by starting, "Took advantage of is the act, too drunk is more of a descriptor of anyone who is willingly too drunk and in the right mindset." This participant’s observation denotes a state of incapacitation where consent cannot be given, whereas "took advantage of" describes an action, implying that the individual may still be perceived in a position to consent.

One female participant expressed her surprise that she even had to relate to her male partner’s consent. To her, gender norms dictated who should provide consent, and who should be asked about being too drunk to provide it:

…I drew on personal experiences. As a woman it is weird to be asked. I’m not going to look at a man and ask if he’s too drunk. I am looking at my own self if I am too drunk, it’s directed towards the female partner (Participant 228).

This response was unique for this one participant. However, it may characterize other survey respondents and so should be taken into account. For this participant, assessing whether someone is too drunk to consent is more about self-assessment than assessment of one’s partner, especially if he is a man.

### Differences Reflecting Perpetration Versus Victimization

When asked to differentiate between “took advantage of while drunk” and “too drunk to consent,” over a third of the participants (38%) interpreted the question by expressing nuances between experiences of perpetration and victimization. One participant stated it explicitly: “The focus of the attention. Took advantage of while drunk is keeping the attention on the perpetrator. Took drunk too consent is focused more on the victim/survivor” (Participant 209). Other participants responded in different variations that the phrases were associated with two different people in different roles, or that one phrase indicated an action while the other a state. For example, a participant reasoned that “took advantage while drunk usually involves another person… While, too drunk to consent is rather a state within your own mental capacity” (Participant 219). Another participant also shared similar sentiments: “Too drunk to consent is you are not able to say yes or no, you were not in the state of mind. Took advantage could be that you are the one taking advantage while drunk” (Participant 200). This participant noted the action in the second phrase as signaling the experiences of perpetration.

Likewise, another participant responded, “Took advantage of thinking **they** are asleep or passed out but too drunk means **you** are awake or moving but won’t remember it the next morning (Participant 225, emphasis added). In this respondent’s view, “took advantage” is something the perpetrator did while seeing someone unconscious, but “too drunk to consent” characterized the victim who couldn’t remember the event after it happened.

For three participants, the different phrases signaled whether or not victimization actually occurred: One participant expressed the variant perspectives between perpetration and victimization, aligning one phrase with the perpetrator’s behavior and the other with the victim’s:

Too drunk to consent sounds like maybe nothing happened because the person was too drunk to consent. Took advantage of while drunk sounds like something did happen without the consent because the person was drunk and unable to think straight (Participant 235).

Another participant echoed this notion: “Took advantage of while drunk is something that happens to you while being too drunk to consent is you being really drunk and nothing has really happened yet, while took advantage of is it already happening.” To these participants, “too drunk to consent” seemed to signal that “nothing happened” because the potential victim was too drunk to consent, whereas “took advantage of” indicated that an act of sexual violence perpetration did occur.

Along somewhat similar lines, another distinction was between an action and a state. One participant differentiated between the two phrases saying, “One implies an action and the other is just a state” (Participant 204). In the words of another participant, “… the latter is sort of a statement before an action, and the other is taking action on that state that the person’s in” (Participant 208). Yet another participant interpreted the two phrases as one presenting a state of mind versus the other describing an action: “Too drunk to consent is you are not able to say yes or no, you were not in the state of mind. Took advantage could be that you are the one taking advantage while drunk” (Participant 200). To all these participants, “too drunk to consent” represented a state while “took advantage of while drunk” represented the action of a perpetrator.

While participants in the study were not specifically asked about sexual violence perpetration or victimization, they related that the different operationalizations reflected either perpetration or victimization. In other words, different phrases for them reflected different types of active (inflicting) or passive (experiencing) states of victimization.

## Discussion

The aim of this study was to examine how young adults interpret two different consent operationalizations of alcohol-involved sexual violence (i.e. "took advantage of while drunk" and "too drunk to consent"), using cognitive interviewing to improve the measurement of sexual violence. To begin with, we found that cognitive interviews were helpful in soliciting a variety of differences and interpretations related to the two phrases. More specifically, we found that participants relied on patterns of behavioral markers to indicate the differences between impairment and incapacitation. Participants also highlighted the ambiguities in consent related to alcohol use, and attributed one phrase more than the other to being incapacitated by alcohol. Finally, respondents claimed the different phrases reflected either a state of sexual violence victimization or an act of perpetration. These themes provide important information on ways to improve the construct validity and therefore increase the accuracy of measurement of alcohol-involved events in sexual violence questionnaires.

### Behavioral Indicators of Consent

A majority of participants in this mixed gender sample agreed that behavioral and emotional markers are indicative of excessive consumption of alcohol and a lack of normal levels of functioning. Specifically, when asked “How can you tell if someone is too drunk to consent?” and “What is the difference between “took advantage of while drunk” and “too drunk to consent?” participants emphasized behavioral indicators of impairment as indicative of alcohol intoxication and a lack of ability to consent. These participants noted language associated with alcohol impairment that may compromise everyday motor and critical thinking skills based on their personal knowledge. In other words, operationalizations that focus on physical cues may be more readily interpretable by respondents. Only a single participant described emotional markers, demonstrating that relying on emotional markers as example of intoxication may not be idea for measurement purposes. The current body of literature indicates that behavioral markers may be indicative of intoxication (Sullivan et al., 2010b). The emphasis of respondents on behavioral indicators may represent a unique phenomenon that has been largely unexplored in the research literature, although it is consistent with the theory of violence measurement (Cook et al., 2011). This theme is also consistent with alcohol myopia theory, in which people may focus on immediate, salient, superficial cues in behavioral symptomatology, rather than covert and embedded cues in emotional impairment. For instance, the physiological cues that usually inhibit decision-making, such as the difficulty walking or talking, and unconsciousness, may be more salient and easy to detect than emotional impairments such as emotional feelings of anger and frustration (Bartholow et al., 2003).

Previous research has highlighted respondents' difficulty in distinguishing between the concepts of impairment and incapacitation (Ullman et al., 2019). To address this, sexual violence questionnaires should include behavioral indicators for both impairment and incapacitation to enhance clarity. Providing examples that align with respondents' individual experiences can improve precision and accuracy, ultimately strengthening the measurement of sexual violence in research. Based on participants’ descriptions, best practices for wording items to identify impairment may include observing behaviors such as slurred speech, speaking loudly, exaggerated gestures, or acting uninhibited. In contrast, according to participants, identifying incapacitation involves more severe indicators, such as not being able to walk, difficulty speaking coherently, or unconsciousness. This is consistent with research on the pharmacological effects of alcohol demonstrating a clear progression of behavioral and physiological changes associated with increasing levels of blood alcohol content (BAC), ranging from mild euphoria and sociability at lower levels to severe impairment, confusion, and even unconsciousness or coma at higher levels (Sullivan et al., 2010a). 

### Complexity in Manifesting Consent

The participants recognized that alcohol can have extreme effects on cognitive functioning and consciousness, such that determining the point at which someone is "too drunk to consent" is a challenging task, as alcohol can cause gradual reduction in coherence, personality, memory, and inhibitions. They recognized the influence of alcohol on consent in both scenarios they were presented with, and generally acknowledged that such consent was at best ambiguous, which is consistent with the broader scientific literature that there is no established standard on how many drinks impair consent (Leone et al., 2022) yet also suggests that participants generally perceive alcohol-involved sex as risky. Nonetheless, some respondents clearly preferred the term "too drunk to consent" over "took advantage of while drunk" to indicate non-consent due to incapacitation. According to their responses, being too drunk to consent was interpreted as manifesting non-consensent, whereas "took advantage of while drunk” still left room for interpretation. Coupled with gendered norms that generally look for consent only on the part of women (Buiten & Naidoo, 2020), these distinctions echo the contrasting 'no means no' versus 'yes means yes' legal standards, where any lack of clear affirmative consent may raise significant concerns about potential sexual violence (Buiten & Naidoo, 2020; Shumlich & Fisher, 2020).

It is noteworthy that in these narratives about incapacitation and consent, participants applied many ambiguous terms such as “probably” and “maybe.” This ambiguity in the language used may reflect the difficulty to distinguish between impairment and incapacitation in the context of sexual consent. Impairment, such as mild intoxication or cognitive challenges, does not necessarily eliminate the capacity to give consent. In contrast, incapacitation, which by definition includes but is not limited to unconsciousness, refers to a state in which an individual’s capacity to make informed decisions is significantly compromised. Using clear language in surveys that applies behavioral indicators specifically tied to incapacitation, where consent cannot be assumed, is essential for consistency and can enhance the accuracy and relevance of survey tools.

While our study did not seek to specifically solicit different gender perspectives, the cognitive interviews with participants revealed some traditional gender norms that may contribute to responses to sexual violence surveys. In particular, one participant expressed surprise that she even had to consider her male partner’s state of consent to sexual activity. This participant's comment reflects social norms in our society whereby it is the woman's job to establish boundaries within sexual activity, protect her own sexuality, and act in charge of consent (Lim & Roloff, 1999). This finding is consistent with research on the importance of using gender-neutral pronouns, including they, to accurately represent and capture the experiences of individuals within questionnaires, and optimize ethical considerations of fairness and respect to foster an egalitarian environment for individuals’ voices to be heard (Suen et al., 2020).

### Perpetration vs. Victimization

Some participants in this study interpreted the question, “What is the difference between ‘took advantage of while drunk’ and 'too drunk to consent’? as reflecting either perpetration (the former) or victimization (the latter), without being prompted to do so. Quite a few of those respondents differentiated between sexual victimization and perpetration based on the action or state expressed in the phrase. In particular, participants concluded that the more passive expression, “[being] too drunk to consent,” indicates the positionality of a victim, meaning an act that is done or forced onto the individual. In contrast, they interpreted the action in the phrase “take advantage of while drunk,” as indicating that the individual is actively engaging in perpetrating sexual violence. Prior research has examined the use of passive voice and active voice to describe sexual violence in media/ new stories (Kanekar et. al., 1981). The current study suggests that the same distinction may apply to questionnaires as well and may elicit different responses based on more active or passive phrasing of sexual violence in the item.

According to psycholinguistic theories, passive sentences (e.g., “being too drunk to consent”) emphasize the role of the victims, making the object, rather than the subject of the sentence more salient. On the other hand, active sentences emphasize the role of the perpetrator, rather than the object, highlighting the subject in perpetuating sexual violence outcomes (Frazer & Miller, 2009). Our findings supports this distinction provided by these theories, and suggests that researchers may want to selectively choose more active or passive (state) descriptions in order to measure sexual violence perpetration or victimization, respectively.

### Limitations

The findings of our study may not be generalizable to other populations across lifespan, because our sample consisted of mostly young adults in college. However, 20% of the sample included community members who were recruited nationally, suggesting that a portion of the overall sample reflected geographical diversity. Additionally, since alcohol-involved sexual violence is most common among college students, a demographic at disproportionately higher risk of sexual victimization (Fedina et al., 2018), they are an appropriate population for a study on measurement of alcohol-involved sexual violence. However, the majority of participants were white, female, and heterosexual, suggesting future research purposefully recruit more inclusive samples to see if the findings replicate across different genders and sexual orientations.

In our research study, we targeted specific questions regarding the different wording in the operationalizations of alcohol-involved sexual violence. However, we could have involved participants in the questionnaire development process by asking them what words or phrasings they would personally use to describe impairment or incapacitation. An example might be, “What words or phrases come to mind when you think about being significantly affected by alcohol and unable to perform certain functions?” This participatory approach empowers participants to contribute to the development of measures that better reflect their lived realities, leading to more valid and relevant research questionnaires. Using a think-aloud protocol (Davison et al., 1997) could have also benefited the study, and should certainly be applied in future studies.

### Directions for Future Research

Future researchers can implement recommendations for phrasing alcohol-involved rape items in questionnaires based on the results from the present study. First, such questions should add specific examples of behavioral markers that are indicative of impairment or incapacitation into the description of the question. Secondly, at least among participants in the present study, the phrase “too drunk to consent” was preferred to “take advantage of while drunk,” as a clearer indication of non-consent due to incapacitation. To diminish the ambiguity between impairment and incapacitation, survey items should give explicit examples of each of them, as noted above. Lastly, researchers should consider the use of action versus a state carefully depending on whether they are attempting to describe perpetration or victimization, respectively.

Furthermore, future research studies could benefit from incorporating participants' sexual experiences and relevant cultural factors in the research question or hypotheses when conducting cognitive interviews. For example, are people with victimization history more sensitive to a particular operationalization of sexual violence than another? Are there any unintended racial interpretations in any of the item phrasings? Different populations may have unique experiences and factors influencing their vulnerability to alcohol-involved sexual violence, and their varied terminology should be recognized. By incorporating these factors and understanding cultural nuances, researchers can develop more robust and contextually appropriate measures for studying alcohol-involved sexual violence.

### Prevention and Policy Implications

The emphasis respondents put on behavioral indicators of impairment suggests this information could also be useful in consent education programs, bystander intervention training, and alcohol-related intervention. From a policy perspective, the findings emphasize that many young people are aware that alcohol consumption can contribute to ambiguous situations and increase the risk of sexual violence. The findings underscore the importance of comprehensive and inclusive approaches to addressing alcohol-involved sexual violence (Leone et al., 2022). This is particularly important since mostsexual and dating violence prevention programs do not discuss alcohol at all in the context of consent (Verbeek et al., 2023). Programs could use the behavioral indicators elicited from participants in the study as examples to consider in their intervention manuals, wiht a focus on safe drinking. Safer Bars interventions are an example of effective alcohol-related violence intervention (Lopez et al., 2015). These interventions focus on creating safer environments in bars and other social settings where alcohol is consumed (Lopez et al., 2015).

## Conclusion

Measuring alcohol-involved sexual victimization is difficult; yet, few studies have used qualitative methods to explore how participants interpret questionnaire items designed to measure alcohol-involved incidents. In this cognitive-interviewing study, behavioral markers were predominantly identified by the majority of the participants in our study as indicators of impaired consent. Incapacitated consent was more easily identified in the phrase “too drunk to consent” than the phrase “took advantage of while drunk.” Additionally, while not asked to distinguish between sexual violence victimization and perpetration in the instructions, over a third of the respondents interpreted the different phrases as indication of either perpetration or victimization, while others associated one phrase with a state of being and the other with an action. Incorporating these interpretations into questionnaire design and item wording could improve the measurement of alcohol-involved sexual violence.
